# Ready-to-Use Multichamber Bags in Home Parenteral Nutrition for Patients with Advanced Cancer: A Single-Center Prospective Study

**DOI:** 10.3390/nu16030457

**Published:** 2024-02-05

**Authors:** María Fernández-Argüeso, Elena Gómez-Bayona, Beatriz Ugalde, Belén Vega-Piñero, Mayra Gil-Díaz, Federico Longo, Rosario Pintor, José I. Botella-Carretero

**Affiliations:** 1 Department of Endocrinology and Nutrition, Hospital Universitario Ramón y Cajal, 28034 Madrid, Spain; arwes.05@gmail.com (M.F.-A.); beatriz.ugalde@salud.madrid.org (B.U.); mariabelen.vega@salud.madrid.org (B.V.-P.); mayra.gil@salud.madrid.org (M.G.-D.); 2 Department of Pharmacy, Hospital Universitario Ramón y Cajal, 28034 Madrid, Spain; egbayona@salud.madrid.org (E.G.-B.); mariarosario.pintor@salud.madrid.org (R.P.); 3 IRyCIS—Instituto Ramón y Cajal de Investigación Sanitaria, Hospital Universitario Ramón y Cajal, 28034 Madrid, Spain; 4 Department of Clinical Oncology, Hospital Universitario Ramón y Cajal, 28034 Madrid, Spain; federico.longo@salud.madrid.org

**Keywords:** home parenteral nutrition, multichamber bag, ready-to-use, cancer, malnutrition, catheter infection

## Abstract

Home parenteral nutrition (HPN) is increasingly prescribed for patients with advanced cancer. This therapy improves free-fat mass, quality of life and survival, but it is not free from complications, especially catheter-related bloodstream infections (CRBSIs). The use of commercial multichamber bags in HPN has not been extensively explored in oncologic patients and their association with complications is not well known. In this prospective cohort study, we included 130 patients with advanced cancer and HPN. We compared the effects of individual compounded bags (n = 87) vs. commercial multichamber bags (n = 43) on complications. There were no differences in any complication, including thrombosis (*p* > 0.05). There were 0.28 episodes of CRBSI per 1000 catheter days in the individual compounded bag group and 0.21 in the multichamber bag group (*p* > 0.05). A total of 34 patients were weaned off HPN, 22 with individual bags and 12 with multichamber bags (*p* = 0.749). Regarding survival when on HPN, the group with individual bags showed a median of 98 days (95% CI of 49–147), whereas those with multichamber bags showed a median of 88 days (95% CI of 43–133 (*p* = 0.913)). In conclusion, commercial multichamber bags for HPN in patients with advanced cancer are non-inferior when compared to individual compounded bags in terms of complications.

## 1. Introduction

Home parenteral nutrition (HPN) is an increasing prescribed therapy for patients with advanced cancer, mostly with gastrointestinal or gynecological tumors [1,2]. Several beneficial effects of HPN have been described in these patients: supplemental HPN may prevent the loss of or even increase free-fat mass in patients with incurable gastrointestinal cancer, and positively impact on quality of life [3,4]; survival was significantly correlated with patient characteristics at HPN start and favorable factors produced an increase in survival [5,6,7]; it was also a relevant palliative therapy for patients with advanced cancer without oral- or enteral-feeding access [8]; it provided important clinical and quality of life benefits; and cost-effectiveness was greater in patients with less severe disease [9,10,11]. Therefore, current nutrition guidelines for patients with cancer recommend the use of HPN whenever necessary, especially in those patients without oral- or enteral-feeding access under active oncologic treatment [12,13,14].

However, HPN is not free from complications, especially the occurrence of catheter-related bloodstream infections (CRBSIs), which can be potentially fatal for these patients [15,16]. Individualized therapy with a multidisciplinary team in centers with HPN management expertise is required as well as careful selection of the catheter type to minimize HPN-related complications [17]. In this regard, there have been several published studies showing that the use of peripheral-inserted central catheters (PICCs) is one of the best choices for delivering HPN, due to the low complications rate, including CRBSI and catheter-related thrombosis [18,19]. In fact, in our setting, single-lumen PICC and Hickman catheters showed the lowest rate of infectious complications [20].

The type and composition of HPN bags have not been extensively explored in oncologic patients, with only few and scattered studies. One recent study with hospitalized patients has shown that the use of multichamber bags was higher among elderly patients with cancer and showed a higher in-hospital mortality [21]. Another one performed in gastric cancer patients after gastrectomy showed that there were no differences in effectiveness and safety measures, the length of hospital stay and costs between multichamber bags vs. individual compounded bags, but the former were more likely to lead to parenteral nutrition-associated liver disease [22]. However, no extensive data have been published in the case of HPN in cancer patients. We aimed to study the effects of individual compounded bags vs. commercial multichamber bags in patients with HPN on the morbidity (mainly infectious complications) and mortality (survival when on HPN) in a prospective single-center cohort.

## 2. Materials and Methods

### 2.1. Patients

One hundred and thirty patients were included in this prospective cohort study. Inclusion criteria were inpatients from the Oncology ward from 2007 to 2022 (both years included) with advanced cancer and intestinal occlusion or sub-occlusion (with or without peritoneal carcinomatosis), regarded as good candidates for HPN because of adequate capacity for self-care and/or a relative or assistance available for home-care. This was evaluated by the Nutrition Unit Team at our center, after a thorough interview with the patients, their relatives and their responsible oncologists. Patients with advanced cancer, both stage IV or other stages with intestinal occlusion or sub-occlusion were amenable to anticancer therapy, and therefore included in the study. Those patients who were not considered candidates for ongoing chemotherapy and/or undergoing only palliative care were excluded from this study, whether or not receiving parenteral nutrition. Those patients with end-stage renal or liver disease, as well as those with advanced dementia were also excluded. The study was conducted in accordance with the Declaration of Helsinki, approved by the Ethics Committee of Hospital Ramon y Cajal (111/15-E2018), and informed consent was obtained from all participants.

### 2.2. Outcomes and Measures

We aimed to identify the effects of HPN in patients with advanced cancer and intestinal occlusion/sub-occlusion regarding prognosis. The primary objective was to compare the effects of individual compounded bags vs. commercial multichamber bags on CRBSI rate. Secondary outcomes included their effects on catheter-related thrombosis and overall survival, as well as to explore other patients’ clinical factor effects. Survival from the starting of parenteral nutrition was recorded, as well as the lines of active antineoplastic treatment. The Eastern Cooperative Oncology Group test (ECOG) was used to assess every patient’s performance status [23].

Anthropometric parameters were measured, body mass index (BMI) calculated, and the percentage of weight loss was also recorded. Estimated daily calorie needs were calculated using the Harris–Benedict equation and multiplied by a factor of 1.2; for the most recent years we used an empirical approach aiming at 20–35 kcal/kg/day. The Malnutrition Universal Screening Tool (MUST) was used for nutritional screening [24]. Blood tests were performed during follow-up as indicated by the treating oncologist, and serum albumin, total cholesterol and lymphocyte count were recorded for the calculation of the Controlling for Nutrition index (CONUT) [25]. Data regarding the composition and type of parenteral nutrition as well as catheter-related data and other complications were recorded and analyzed as indicated below.

### 2.3. Composition and Infusion of Parenteral Nutrition

The composition of parenteral nutrition followed current guidelines [13]. Our Pharmacy Department uses both individual compounded parenteral nutrition and commercial multichamber bags (Smofkabiven^®^, Fresenius Kabi, Bad Homburg, Germany; and Olimel^®^, Baxter, Deerfield, IL, USA). Patients received either of them, or both, during their follow-up, as required. The choice of the type of bag was not randomized, but was indicated by the patients’ needs and the characteristics of the compounded bags. The latter were used whenever the patients’ needs for macro- and micronutrients were fulfilled by the available bags in the Hospital Pharmacy at the time. For those patients with high volume and/or electrolyte needs, individual bags were preferred. We aimed at 20–35 kcal/kg/day, with a proportion of 3–6 g/kg/day for glucose, 1.0 g/kg/day for amino-acids and less than 1.0 g/kg/day for lipids, with 7–10 g/day of essential fatty acids. Vitamins and trace elements (Cernevit^®^, Baxter, Deerfield, IL, USA, and Supliven^®^, Fresenius Kabi, Bad Homburg, Germany, respectively) were also added for those patients who were unable to take these supplements orally. These patients were prescribed a commercial multivitamin covering a standard adult’s requirements (Supradyn^®^, Bayer, Leverkusen, Germany, or Multicentrum^®^, Haleon, Surrey, UK).

At our center, there is a specialized team that includes an Intravenous Therapy Unit (ITU). All the protocols are designed and all the personnel are adequately trained in order to give the best care to patients with parenteral nutrition, both in hospital and at home, not only regarding their artificial nutrition but also their venous accesses.

All patients and, if needed, some of their relatives, were appropriately trained for adequate manipulation of both the central catheter and parenteral nutrition infusion pumps and connections, including the types of alarms and ways to solve the problems. Several sessions were conducted before hospital discharge so as to ensure proper autonomy and security of all procedures regarding the management of HPN. They were provided with written instructions in a booklet with all necessary information and a contact phone number was also available during weekdays for any question they may have. All patients, whatever the type of parenteral nutrition bag or type of catheter, received full education and information and only differed regarding the different preparation of the parenteral nutrition bag and the specific care of the different types of venous access. After hospital discharge, HPN was infused on an intermittent schedule, primarily at nighttime. Patients were followed up every 2 weeks the first two months, and every 1–3 months thereafter.

### 2.4. Central Venous Catheters and Their Related Complications

The choice of the venous catheter was not randomized but based on the patient’s responsible physician, always taking into account the underlying disease, the expected duration of HPN, and the possibility of a safe procedure for obtaining a venous access. Ports and Hickman catheters were implanted at the Intervention Radiology Department, with fluoroscopy guidance and local anesthesia. PICCs were implanted at the ITU clinic, with local anesthesia, and with ultrasound guidance. Maximal barrier precautions were maintained for all catheter insertions. Multilumen catheters were chosen when patients needed additional medication for long-term infusion in addition to parenteral nutrition.

Local catheter infections were defined as an exit-site infection (defined as redness, swelling, tenderness, with an erythema of more than twice the diameter of the catheter), tunnel infection, or pocket infection. CRBSI was confirmed by isolation of the same microorganism from quantitative pair blood cultures from the catheter lumen and the blood peripherally drawn, and after catheter tip-removal with positive semi-quantitative cultures (roll-plate Maki technique). In the case of negative semi-quantitative culture, quantitative culture with sonication was also performed. Venous thrombosis was evaluated using ultrasonography (but not routinely if asymptomatic) when compatible symptoms and signs were reported by patients.

### 2.5. Statistical Analysis

Sample-size analysis was performed with the online software Cleveland Clinic Sample Size Calculator [26] for a non-inferiority study that compares the use of individual compounded parenteral nutrition bags vs. commercial multichamber bags, regarding our previous reported rate of infectious complications [20]. With α = 0.05, β − 1 = 0.8, and a mean rate of catheter-related infection of 0.4 per 1000 days of catheter use, a total of 108 patients (with a 2:1 proportion between groups) is required to exclude a difference of more than 10% excess of risk.

Results are expressed as means ± SD unless otherwise stated. The Kolmogorov–Smirnov statistic was applied to continuous variables. Logarithmic or square root transformations were applied as needed, to ensure a normal distribution of the variables. Comparisons between the different groups at baseline were performed by independent *t* test for continuous variables or the Mann–Whitney U test for non-normal distributed variables, and by χ^2^ test or Fisher’s exact test for discontinuous variables. Survival was analyzed by Kaplan–Meier estimator, and the log rank test and multivariate Cox proportional hazards test and hazard ratios (HR) calculated. Analyses were performed using SPSS 18 (SPSS Inc., Chicago, IL, USA). *p* < 0.05 was considered statistically significant.

## 3. Results

One hundred and thirty patients with advanced cancer and intestinal occlusion or sub-occlusion on HPN were included in this prospective cohort from 2007 to 2022 (both years included). The underlying types of neoplasia are shown in Table 1. The majority of tumors were gastrointestinal in origin, with a clear predominance of gastric and colon cancer. The second most frequent neoplasia was of gynecological origin, mostly ovarian cancer (Table 1).

Eighty-seven patients had individual compounded parenteral nutrition bags, whereas forty-three had commercial multichamber bags (nineteen patients needed a change to individual compounded ones for some short periods of time). Demographic and clinical variables of the patients depending on the type of HPN bag are shown in Table 2. There were no differences in any clinical or prognostic variables, including CRBSI or thrombosis (Table 2).

There were 0.28 episodes of CRBSI per 1000 catheter days in the individual compounded bag group and 0.21 in the multichamber bag group (*p* > 0.05). Pathogens found in the former group were one Staphylococcus epidermidis (after 151 days of HPN use) and two Coagulase-negative Staphylococci (after 73 and 109 days of HPN use, respectively), whereas in the latter group a Staphylococcus epidermidis was found after 62 days of HPN use.

When we compared the venous access for parenteral nutrition, we found that ports had the highest rate of CRBSI (1.13 per 1000 catheter days) compared to Hickman or PICC (0.23 per 1000 catheter days, *p* = 0.037). When we analyzed the association of the number of lumen of the employed catheters, we found that multilumen Hickman and PICC had higher risk of infection than single-lumen ones (*p* = 0.039). When nutritional data (body weight, BMI, serum albumin, and CONUT index both at baseline and at follow-up) were compared in those patients with and without CRBSI, no significant results were obtained (*p* > 0.05 for all comparisons).

Only two episodes of venous thrombosis were found which took place in the group of HPN with individual compounded bags. Both occurred with two-lumen Hickman catheters and after long-term use of HPN (390 and 786 days, respectively).

A total of 34 patients were weaned off parenteral nutrition, 22 with individual compounded bags and 12 with commercial multichamber bags (*p* = 0.749). The tumor stages of these patients did not differ from those patients who stayed on HPN (50.0% vs. 52.1%, *p* = 0.835), and neither did the type of neoplasia (GI 68.8%, Gine 20.8%, Other 10.4% vs. GI 67.6%, Gine 20.6%, Other 11.8%, *p* = 0.977). Patients who were weaned off HPN presented higher BMI (*p* = 0.048), serum albumin concentrations (*p* = 0.012) and CONUT score (*p* = 0.001) at the end of follow-up, and they received fewer chemotherapy lines before starting HPN (*p* = 0.045).

**Figure 1:**
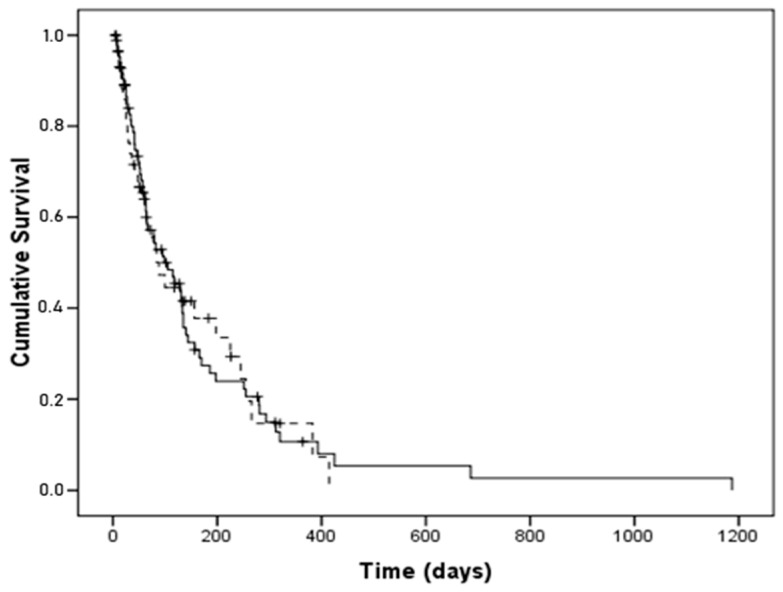
Kaplan–Meier survival curves for patients with individual compounded bags (continuous line) vs. commercial multichamber bags (dashed line). There was no significant difference (χ^2^ log rank test = 0.012, *p* = 0.913).

Regarding survival when on HPN, the group with individual compounded bags showed a median of 98 days with a 95% CI of 49–147, whereas the group with commercial multichamber bags showed a median of 88 days with a 95% CI of 43–133 (χ^2^ log rank test = 0.012, *p* = 0.913) (Figure 1).

**Figure 2:**
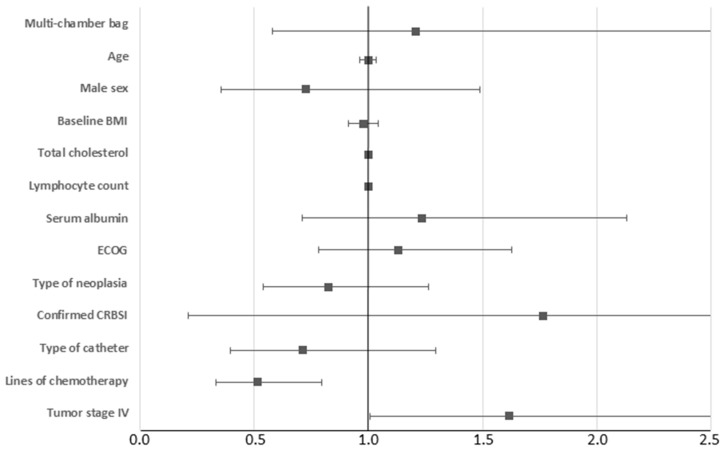
Multivariate analysis with the Cox proportional hazards test. Squares show the HR for each variable and horizontal error bars show the 95% CI of the HRs.

Finally, a multivariate analysis was performed with the Cox proportional hazards test in which age, gender, type of neoplasia (gastrointestinal, gynecologic or other), type of HPN bag (individual compounded or commercial multichamber), type of catheter (PICC, tunneled or port), baseline ECOG and lines of chemotherapy after the beginning of HPN were introduced as covariates. In this model (χ^2^ = 15.333, *p* < 0.001), only the number of lines of chemotherapy that patients received after the initiation of HPN was a significant beneficial associated variable (HR = 0.649, 95% CI = 0.498–0.845 for mortality). A second model (χ^2^ = 14.697, *p* < 0.001), with baseline BMI, serum albumin, total lymphocytes, total cholesterol, CRBSI and catheter thrombosis as covariates, was analyzed. Again, the only associated variable was the number of lines of chemotherapy that patients received after the initiation of HPN (HR = 0.514, 95% CI = 0.333–0.794 for mortality). A third model also included the tumor stage (stage IV vs. other stages) and this resulted also to be an associated variable, with survival (HR = 1.616, 95% CI = 1.008–2.591 for mortality) (Figure 2).

## 4. Discussion

In this large prospective study, we have shown that commercial multichamber parenteral nutrition bags were non-inferior to individual compounded bags regarding infectious complications for patients with advanced cancer who were at home. To date, there have been only scattered studies regarding this issue, and none of them focused on oncologic patients with advanced cancer and intestinal occlusion or sub-occlusion.

A recent study including 60 patients on HPN with chronic intestinal failure with multichamber parenteral nutrition bags and 45 with individual compounded bags, with CRBSI rates of 0.51/1000 catheter days and 0.39/1000 catheter days respectively, showed no statistical difference, in accordance with our results [27]. However, this study was not specifically and solely focused on patients with advanced cancer, like our study. Another old and small study from our country with only eight patients on HPN with multichamber bags showed that the BMI and the fat-free mass index increased during the treatment, the Karnofsky index was maintained or increased, and four episodes of CRBSI were observed with an incidence of 0.85/1000 catheter days [28]. This study also underlined the safety of this type of bags for patients on HPN, but the limitations were the small sample size, lacking a comparable cohort with individual compounded bags, and the fact that it was not specifically focused on patients with advanced cancer.

A retrospective study with 64 gastric cancer patients who underwent gastrectomy in a tertiary teaching hospital receiving parenteral nutrition after the operation showed that there were no significant differences between the use of multichamber bags vs. individual compounded bags in terms of BMI preservation, metabolic complications, the length of hospital stay and costs [22]. Only total bilirubin and direct bilirubin were both significantly higher in the former. However, this study was carried out in hospitalized patients, with parenteral nutrition only after surgery, and it did not include patients on HPN.

Another study with a large population of hospitalized patients (n = 84,564 patients) but with a small proportion of multichamber bags (21.1%) showed that the latter, when not receiving addition (only 6.3%), were associated with less incidence of CRBSI and less cost [29]. However, when multichamber bags had addition, there were no differences with individual bags in terms of CRBSI incidence, although the analysis showed lower costs. This was a retrospective study, and with adult inpatients, so data cannot be generalized to oncologic patients with HPN [29].

The rates of CRBSI were similar in our study between multichamber bags and individual compounded bags, and we found that the type of catheter was the most important factor associated with the risk of infectious complications, as previously shown [30]. Therefore, it is important to analyze the venous access employed for parenteral nutrition in order to evaluate previous studies reporting the incidence of infectious complications when comparing the type of bag or the composition of parenteral nutrition. This, if not taken into account, may bias the results of the rates of CRBSI in those studies.

Albeit there are theoretical advantages of premixed multichamber bags over individual compounded bags for parenteral nutrition, regarding less cost [21], decreased compounding time, reduced chance for error, and lower incidence of bloodstream infections in hospitalized patients [31], they may not be appropriate for all patients [32,33]. One study showed higher electrolyte disturbances with multichamber parenteral nutrition bags [34], so this may have been a drawback for oncologic patients who may theoretically need more changes in the composition at follow-up. However, this was not the case in our study and, in fact, metabolic complications did not differ between those patients on commercial multichamber bags vs. individual compounded bags.

Survival of patients with advanced cancer on HPN was shown to be increased in a study with 125 patients undergoing palliative care when compared with only hydration [7]. The median overall survival was 4.3 months in those patients on HPN vs. 1.5 months in those with only hydration (*p* < 0.001). The multivariate analysis of the risk factors for mortality showed that not receiving HPN accounted for the strongest one [7]. Another study with 153 advanced cancer patients who received HPN also analyzed survival: 35% survived for 6 months, 27% for 1 year, 18.9% survived 2 years, and 3.9% survived for the 7 years of the follow-up. Hospitalization rate was not significantly different from the noncancer population, results that highlight the relevant role of HPN in this population [8].

A previous prospective cohort study with 761 adult malnourished cancer patients on HPN aimed to investigate clinical characteristics, predictive factors, and overall survival [5]. The authors found that the predictors showing significant association with decreased survival were the modified Glasgow Prognostic Score (1 and 2 scores), weight loss (>15%) in the 3 months before HPN start, and tumor advanced stage, while protective factors of survival were higher performance status, albumin level, oral protein intake, and BMI at HPN start. Furthermore, patients in the group receiving supplemental parenteral nutrition and active chemotherapy survived significantly longer [5], a result in accordance with our present data.

Conversely, other studies have yielded different and heterogeneous prognostic factors associated with survival in patients with advanced cancer on HPN. Keane et al. reported that performance status, prognostic scoring, and parenteral nutrition requirements regarding volume and potassium may predict survival in patients with advanced cancer receiving HPN [35]. Bozzetti et al. proposed and validated a nomogram to predict survival in incurable cancer patients on HPN [36]. They found that Glasgow and Karnofsky scales for performance, tumor site and spread were significant prognostic factors. Pasanisi et al. showed that survival differed widely and could be predicted only to a limited extent from initial values of serum albumin and Karnofsky performance status [37]. The heterogeneous nature of the different factors that may be associated with the survival in patients with advanced cancer on HPN may reflect differences in the inclusion criteria of the different studies, as well as different methodology when recording the clinical data and HPN-related complications. Furthermore, the inclusion of various cancer types, clinical stages and types of treatment made the survival analysis not a reliable one in most of the aforementioned studies.

Although survival analysis was a secondary outcome of our study and therefore the sample size was a small one in order to accomplish this objective, we found that the number of lines of chemotherapy that patients received after the initiation of HPN was a prognostic factor associated with survival, and tumor stage IV was associated with poorer prognosis. Also, those patients who were weaned off parenteral nutrition received fewer chemotherapy lines before starting HPN, probably reflecting the fact that an early stage of the treatment timeline may be associated with a better prognosis.

The advantages of our study are the long-term follow-up of our cohort, and the homogeneous education and care of the patients guided by a specific protocol and unit. However, several limitations have to be acknowledged: first, the allocation to the type of parenteral nutrition bag, as mentioned above, was decided on the patients’ needs for macro- and micronutrients and whether or not these were fulfilled by the available bags in the hospital pharmacy at the time; however, as the different allocation did not result in any differences in metabolic or other complications, we do not think it biased the results. Second, the type of venous catheter was not randomized, but rather indicated by the patients’ responsible doctors’ choice. Third, the sample size and the inclusion of various cancer types, clinical stages and types of treatment made the survival analysis not a reliable one, as occurred with the previously discussed studies. However, we have to keep in mind the fact that survival analysis was a secondary outcome of our study. Therefore, it is necessary to conduct multicenter studies with larger populations and precise methodology, in order to find results that are more consistent.

## 5. Conclusions

The use of commercial multichamber bags for HPN in patients with advanced cancer and intestinal occlusion or sub-occlusion are non-inferior compared to individual compounded bags in terms of complications and may be more convenient and with fewer associated costs.

## Figures and Tables

**Table 1:** Types of underlying neoplasia of the included patients (n = 130).

**Gastrointestinal neoplasia**	**89**
Esophageal cancer *	8
Gastric cancer with PC or obstruction	38
Pancreatic cancer with PC or obstruction	9
Intestinal adenocarcinoma with PC	3
Neuroendocrine tumor with obstruction	4
Appendicular carcinoma	1
Colorectal cancer with PC	25
Rectal cancer with radiation enteritis	1
**Gynecological neoplasia**	**28**
Cervix carcinoma with PC	1
Breast cancer with PC	5
Ovarian cancer with PC	19
Fallopian tube carcinoma with PC	2
Endometrium cancer with PC	1
**Others**	**13**
Bladder cancer with PC or fistula	4
Bone sarcoma with PC	1
Lung cancer with GI involvement	1
Head and neck cancer *	3
GVHD after leukemia	4

Text in bold indicate subtotal for the specific subgroup. * Enteral nutrition and/or prosthesis not feasible. PC: peritoneal carcinomatosis. GI: gastrointestinal. GVHD: graft vs. host disease.

**Table 2:** Clinical and prognostic variables according to the type of HPN bag *.

	Individual Compounded (n = 87)	Commercial Multichamber (n = 43)
Age (y)	58 ± 11	59 ± 13
Female gender	57 (66)	30 (69)
Time on HPN (days) ^‡^	64 ± 113	64 ± 126
Duration > 6 months	70 (80)	29 (68)
Bags infused per week (n)	6.9 ± 0.6	7.0 ± 0.0
Supplied energy (Kcal/kg/day)	29.4 ± 7.9	28.7 ± 7.6
Body weight (kg)	53 ± 13	55 ± 14
BMI (kg/m^2^)	20.2 ± 4.4	21.1 ± 5.5
Serum albumin (g/dL)	2.7 ± 0.7	2.7 ± 0.5
Total lymphocytes (n/10^3^)	1.2 ± 0.7	1.3 ± 0.8
Total cholesterol (mg/dL)	205 ± 205	163 ± 47
CONUT index (score)	7.2 ± 2.4	8.2 ± 2.8
MUST index (score)	1.9 ± 0.3	1.9 ± 0.3
ECOG index (score)	1.7 ± 0.9	1.7 ± 0.8
Gastrointestinal neoplasia (n)	62 (71)	27 (63)
Stage IV (n)	29 (48)	14 (52)
Gynecologic neoplasia (n)	18 (21)	10 (21)
Stage IV (n)	14 (78)	7 (70)
Other type of neoplasia (n)	7 (8)	6 (14)
Stage IV (n)	3 (43)	3 (50)
Lines of chemotherapy before HPN (n)	1.8 ± 1.8	1.3 ± 1.4
Lines of chemotherapy after HPN (n)	2.5 ± 2.0	1.9 ± 1.6
PICC catheter (n)	56 (65)	33 (78)
Tunneled catheter (n)	9 (10)	2 (5)
Ports (n)	22 (25)	7 (17)
Multilumen catheter (n)	12 (19)	7 (21)
Suspected CRBSI (n)	8 (9.2)	2 (4.7)
Confirmed CRBSI (n)	3 (3.4)	1 (2.3)
Thrombosis (n)	2 (2.3)	0 (0)
Severe metabolic complications (n) ^†^	1 (1.2)	0 (0)

Data are means ± SD or n (%) ^‡^ except for time on HPN, which are medians ± interquartile range. BMI: body mass index. CONUT: controlling for nutrition index. MUST: malnutrition universal screening tool. ECOG: Eastern Cooperative Oncology Group test. CRBSI: catheter-related blood-stream infection. * No significant differences were found in any variable. ^†^ Severe metabolic complications that needed admission.

## Data Availability

Restrictions apply to the availability of the data generated or analyzed during this study to preserve patient confidentiality or because they were used under license. The corresponding author will, on request, detail the restrictions and any conditions under which access to some data may be provided.
